# Evaluation of the Interface Strength in the Abaca-Fiber-Reinforced Bio-Polyethylene Composites

**DOI:** 10.3390/polym15122686

**Published:** 2023-06-15

**Authors:** Faust Seculi, Francesc X. Espinach, Fernando Julián, Marc Delgado-Aguilar, Pere Mutjé, Quim Tarrés

**Affiliations:** LEPAMAP-PRODIS Research Group, University of Girona, 17003 Girona, Spain; faust.seculi@udg.edu (F.S.); fernando.julian@udg.edu (F.J.); m.delgado@udg.edu (M.D.-A.); pere.mutje@udg.edu (P.M.); joaquimagusti.tarres@udg.edu (Q.T.)

**Keywords:** biocomposites, BioPE, abaca fibers, interface strength, intrinsic tensile strength, micromechanics

## Abstract

Bio-based polymers, with any of their constituents based on nonrenewable sources, can answer the demands of society and regulations regarding minimizing the environmental impact. The more similar such biocomposites are to oil-based composites, the easier the transition, especially for companies that do not like the uncertainty. A BioPE matrix, with a structure similar to that of a high-density polyethylene (HDPE), was used to obtain abaca-fiber-reinforced composites. The tensile properties of these composites are displayed and compared with commercial glass-fiber-reinforced HDPE. Since the strength of the interface between the reinforcements and the matrix is responsible for the exploitation of the strengthening abilities of the reinforcements, several micromechanical models were used to obtain an estimation of the strength of the interface and the intrinsic tensile strength of the reinforcements. Biocomposites require the use of a coupling agent to strengthen their interface, and once an 8 wt.% of such coupling agent was added to the composites, these materials returned tensile properties in line with commercial glass-fiber-reinforced HDPE composites.

## 1. Introduction

Material sciences have a relevant role in the development of new materials that contribute to green design, sustainable design, circular economy, and other design frameworks based on minimizing the impact of artifacts on the environment [1,2,3]. Life cycle assessment (LCA) is one of the methodologies used to evaluate such impact, accounting for the use of raw materials, manufacturing technique distribution and transport, and use and disposal or recycling of the artifacts. LCA has to be performed on products; the materials and techniques used to obtain the products affect noticeable LCA results [4,5]. Materials such as bio-based polymers or natural fiber reinforcements can show their competitiveness compared to oil-based polymers and manmade mineral fibers that spend huge amounts of energy during their fabrication. In any case, all these advantages cannot be significant if bio-based materials are unable to show mechanical properties similar to those of commercial materials and can be processed under the same or similar conditions [6]. The authors aim to study the strength of the interface between abaca fibers and bio-based polyethylene and evaluate whether it is strong enough to compete with oil-based matrix composites.

Natural fibers as reinforcement for polymers have several advantages over mineral fibers such as glass fiber. Natural fibers are lighter than glass fiber, and this affects the amount of energy needed to transport such materials; in addition, if the materials are used by the transport industry, they lighten the vehicles, and such vehicles can decrease their fuel consumption [7,8,9,10,11]. Ashby diagrams show that natural materials can reach Young’s moduli similar to oil-based polymers at lower densities. This is the case of bamboo, with a density of 0.78 to 0.54 g/cm^3^ and Young’s modulus of 35.45 GPa compared with an HDPE with Young’s modulus of 0.85 GPa and a density of 0.95 g/cm^3^ [12]. Thus, the specific properties (densities: 1.2–1.6 cm^3^) of natural-fiber-reinforced polymers could be higher than those of materials with the same polymer reinforced with glass fibers (density: 2.48 g/cm^3^) [13,14]. Moreover, natural fibers are not harmful and can be manipulated easier than glass fiber [15]. In addition, natural fibers are less abrasive than glass fiber and their use increases the lifespan of the equipment [15]. Nonetheless, natural fibers show some drawbacks compared to mineral fibers. Natural fibers are lignocellulosic materials, and the temperature of degradation of cellulose is around 200 °C, limiting the use of the fibers as reinforcement to plastics that can be transformed at this or lower temperature [16]. In any case, polymers such as polyethylene and polypropylene that are widely used by the industry, alone or reinforced with glass fiber, are within these parameters. Another major drawback of natural fibers is their hydrophilicity, which limits their compatibility with hydrophobic matrices such as polyolefins. This problem has been known for a long time since the time the first natural-fiber-reinforced thermoplastic composites were tested [17,18,19]. Fortunately, there are several fiber treatments or additives that can solve or minimize this problem [20,21,22,23,24]. Moreover, the intrinsic properties of natural fibers show higher scatter than manmade fibers, and such properties are impacted by the origin of the fibers or the climatology of the harvesting season. Nevertheless, natural fibers have proven competitive as reinforcement for oil-derived plastics, reaching mechanical and thermal properties similar to those of glass-fiber-reinforced commodities [25,26,27].

A new stage in the search for totally bio-based composites is the substitution of oil-based matrices with bio-based ones. There are several options, and the list of bio-based polymers is increasing. Polymers such as poly (lactic acid), polybutylene adipate terephthalate (PBAT), bio-polyamide (BioPA), BioPolyethylene (BioPE), and starch-based polymers are some of the most relevant [28,29,30,31,32,33]. To promote their use by the industry, the similarity of biopolymers to commercial polymers is important. In this sense, the industry is using huge amounts of high-density polyethylene (HDPE), which is very similar to BioPE. Thus, any artifact made with HDPE can be made with BioPE with no or few changes in the equipment or process parameters [34]. The main drawback of BioPE is its cost, which is higher than that of HDPE. This is related to its processing, and future developments and scale economies can reduce this gap. BioPE has shown a lower environmental impact than HDPE, but some authors stress the social impact [34,35]. BioPE is derived from the dehydration of fuel-ethanol which is based on sugar cane, starch crops, sugar beet, or lignocellulosic materials. Feedstock that can be used to nourish persons or animals indeed has social implications and impact. Thus, it is important to further develop the methodologies that allow the obtention of BioPE from lignocellulosic sources such as agroforestry waste [36].

Natural fibers are more usually used as reinforcement for polymers, including abaca, kenaf, hemp, flax, ramie, jute, and bamboo [7,33,37,38]. These fibers show intrinsic tensile strengths ranging from 220 to 1500 MPa. It must be taken into account that E glass fibers and S glass fibers have 3800 and 45,810 MPa tensile strengths, respectively [13,14]. Thus, the strengthening ability of glass fiber per mass unit is higher than that of natural fibers. In the case of Young’s modulus, natural fibers show moduli in the range from 12 to 128 GPa, and the moduli of glass fibers range from 73 to 85 GPa [13,14]. Despite the differences between the intrinsic properties of natural fibers and glass fibers, the former has proven competitiveness compared to the latter. Abaca fibers, with tensile strengths ranging from 400 to 980 MPa, and Young’s moduli, tested from 12 to 42 GPa, depending on their precedence and treatments are reinforcements that are widely used and studied in the literature because some authors refer to such fibers as the strongest of natural fibers, exhibiting high resistance to decomposition under salt water and better tensile properties than manmade fibers such as nylon or rayon [37]. The main provider of abaca fibers is the Philipines, which already established commercialization circuits of abaca fibers for paper pulp or cordage. Moreover, abaca-fiber-reinforced composites showed tensile strength ranging from 70 to 365 MPa [13,14]. These values depend noticeably on the percentage of fiber, the matrix, the morphology of the fibers, especially its aspect ratio (ratio between the length and diameter), and the orientation of the fibers against loads.

Natural-fiber-reinforced biopolymers have grown in importance in the literature in the last few years. The substitution of oil-based matrices for natural-fiber-reinforced composites is the expected evolution towards more sustainable materials. BioPE has called the attention of several researchers. Garcia-Garcia et al. obtained biocomposites from BioPE and peanut shells [39]. They used 3 wt.% of coupling agents to increase the strength of the interface, but their composites showed tensile strengths lower than those of the matrix. On the other hand, Young’s and flexural moduli, as well as the flexural strength of the composites, increased regarding the matrix. The authors centered their work around the effect of the coupling agents but did not experiment with variable coupling agent percentages. Bazan et al. evaluated the tensile properties of a BioPE reinforced with wood flour, coconut fibers, or basalt fibers, preparing coupled and uncoupled composites [40]. In this case, the percentages of reinforcement were 6 and 12 wt.%. The authors found little impact of the reinforcements for uncoupled composites but a positive correlation between fiber content and tensile properties for the coupled composites. Coupled composites exhibited tensile strength higher than that of basalt fiber-reinforced composites. The authors used some micromechanical models to preview the properties of the composites, finding that the properties previewed by the models noticeably overrated or underrated experimental values. In another research, Bazan et al. evaluated the mechanical thermal and aging properties of wood fiber, flax, coconut, and basalt fiber-reinforced BioPE [41]. Their conclusions were similar to those of the previous article, adding the decrease in mechanical properties after immersing the specimens in water studying the water uptake and reaching saturation of the samples. Serra-Parareda et al. used BioPE to evaluate barley-reinforced composites [42]. The authors used 6 wt.% of coupling agent and found a relation between coupling agent content and tensile strength. The authors used the Kelly and Tyson equation to evaluate the strength of the interface [43]. The interface was rated as strong, and tensile strength was positively correlated with the percentage of barley fibers. Aguado et al. evaluated the tensile properties of rapeseed-fiber-reinforced BioPE [44]. The authors treated the fibers under different conditions, using a mill, a mechanical treatment, and a thermomechanical treatment. These treatments affected the yield of the raw material and changed slightly the chemical composition of the fibers and their morphology. The authors found that the treatments had a noticeable effect on the mechanical properties of the composites and the morphology of the fibers. While the thermomechanical treatment returned better properties than the mechanical treatment, the slight differences were difficult to justify in comparison to the yield of both processes. The authors evaluated the strength of the interface with the Kelly and Tyson equation, finding that the use of 6 wt.% of coupling agents created a strong interface. Tarres et al. evaluated the evolution of Young’s modulus of corn-stover-reinforced BioPE [45]. The researchers found a positive correlation between coupling agent content and reinforcement content over the mechanical properties of the composite. The authors also evaluated the mean orientation angle of the reinforcements, showing that the orientation was not random. Almeida Barbalho et al. evaluated sugar-cane- and curaua-reinforced BioPE composites [46]. The researchers stated the importance of fiber treatment and the use of coupling agents to obtain a strong interface. The authors of the present work studied the intrinsic mechanical properties of abaca fibers by single fiber test, obtaining a maximum intrinsic tensile strength of 589 MPa, well inside the values published in the literature [47]. In another study, the authors evaluated the evolution of Young’s modulus of abaca-fiber-reinforced BioPE against reinforcement content [48]. They found a positive correlation between the percentage of reinforcement and the stiffness of the composites. They could not find a correlation between coupling agent content and stiffness.

The literature points out the value of totally bio-based composites and evaluates their costs, environmental impact, and thermal and mechanical properties. To date, the number of publications devoted to totally bio-based composites is lower than that of natural-fiber-reinforced oil-derived matrices, but it is growing. Revised literature shows the positive correlation between natural fiber contents and mechanical properties, as well as the importance of a strong interface. Nonetheless, authors use only a micromechanical model to evaluate such interface strength. In this paper, the authors evaluate the tensile properties of coupled and uncoupled abaca-fiber-reinforced BioPE composites. The authors compare these mechanical properties with those of glass-fiber-reinforced HDPE materials. Finally, the authors propose four methods to evaluate the strength of the interface based on a modified rule of mixtures [49,50], the evaluation of the length and interface factor [51], the Kelly and Tyson equation [43] with the solution proposed by Bowyer and Bader [52], and the Kelly and Tyson equation. The authors discuss the limitations of the methods and the different interpretations in the cases of contradictory results. 

## 2. Materials and Methods

### 2.1. Materials

The main composites were prepared with a bio-based polyethylene (BioPE) matrix SHA7260 kindly supplied by Braskem (Sao Paulo, Brazil). These composites were reinforced with abaca fibers (AF) provided by CELESA (Tortosa, Spain). Abaca strands were received and cut at 700 mm, manually cut to 100 mm and then chopped to 5 mm in a mill. Then, the strands passed through a hammer mill equipped with a 6 mm screen. The fibers were characterized in a previous work and contained 72.7% of cellulose, 14.6% of hemicellulose, 8.9% of lignin, 2.9% of extractives, and 0.9% of ashes [47]. The density of the AF and BioPE was 1.45 g/cm^3^ and 0.95 g/cm^3^, respectively.

A Fusabond^®^ MB100D 0.9% coupling agent based on polyethylene functionalized with maleic acid (MAPE), commercialized by DuPont (Wilmington, DE, USA), was used to increase the compatibility between the matrix and AF and obtain stronger interfaces.

To compare the mechanical properties of bio-based composites with glass-fiber (GF)-reinforced oil-based composites, a second batch of materials was prepared using an HDPE 5226EA INEOS matrix provided by Suministros Plásticos Europeos (Barcelona, Spain) with a density of 0.95 g/cm^3^. This matrix was reinforced with E glass fibers by Vetrotex (Chamberly Cedex, France) that was provided by Maben, S. L. (Banyoles, Spain). These fibers showed an average length of 3.3 mm before mixing or mold injection and a density of 2.45 g/cm^3^. A sizing agent was added to increase its compatibility with the matrix.

### 2.2. Composite Fabrication

BioPE, abaca fibers, and MAPE were mixed in an intensive G5S Gelimat kinetic mixer by Draiswerke (Mahaw, NJ, USA). The phases were mixed in the equipment for 2 min at 3000 rpm until a 190 °C discharge temperature was obtained. The blends were discharged from the kinetic mixer, cooled down, and pelletized in a hammer mill to 5 mm particles, able to be mold-injected. Composites with a 30 wt.% AF and 0, 2, 4, 6, 8, and 10 wt.% MAPE contents were prepared to evaluate the impact of the coupling agent on the tensile properties of the composites. Previous studies show that the percentage of coupling agent that returns higher tensile strength is similar for different percentages of reinforcement [53,54]. The authors conducted the study for a 30 wt.% AF content because this percentage has been used previously, and because it returns the coupling agent contents applicable to percentages of reinforcement in the range from 10 to 50 wt.% [53]. Once it was established that 8 wt.% of MAPE returned the highest tensile strength, composites with this MAPE content and 20, 40, and 50 wt.% AF were prepared. 

HDPE composites, reinforced with 10, 20, and 30 wt.% GF contents were prepared in a Brabender^®^ plastograph internal mixing machine. The equipment worked at 20 rpm for 10 min at a temperature of 180 °C. Obtained blends were cooled down and pelletized. 

The authors used different equipment to prepare AF- and GF-based composites because the Gelimat Kinetic mixer subjects the reinforcements to attrition that results in fiber shortening. While the shortening is acceptable for natural fibers, it is not so for AF. Brabender^®^ plastograf allowed a milder treatment that minimized such fiber shortening.

The evaluation of the fiber volume fraction was obtained from the following equation:

(1) $$V^{F}=\frac{w^{F}\cdot \rho^{F}}{w^{m}\cdot \rho^{F}-w^{F}\cdot \rho^{m}},$$

where $V^{F}$ is the fiber volume fraction, $w^{F}$ and $w^{m}$ are the masic fractions of reinforcement and matrix, respectively, and $\rho^{F}$ and $\rho^{m}$ are the densities of the reinforcement and the matrix, respectively.

### 2.3. Dog Bone Specimen Obtention and Tensile Test of the Specimens

All residual humidity was eliminated from the pellets by storing them in a stove at 80 °C 24 h before their mold injection. Dry pellets were mold-injected in a steel mold with cavities corresponding to ASTM D638 tensile (dog bone) specimens. The equipment was a Meteor 40 by Mateu and Sole (Barcelona, Spain). The machine was operated to reach 190 °C in the nozzle and 175 to 180 °C in the barrel. All temperatures were carefully monitored to prevent overheating that can affect the structure of the lignocellulosic reinforcements. The filling pressure was 117.7 bar, and maintaining pressure was 24.5 bar. At least ten specimens of any of the formulated composites were obtained. The mold was maintained at 70 °C. This temperature favors the crystallization of the matrix. Cooling down inside the mold lasted around 30 s.

Specimens were stored in a conditioning chamber at 23 °C and 50% relative humidity for 48 h before their tensile testing. The specimens were tensile tested under STM D638 specifications in an Instron^®^ 1122 Universal testing machine. This equipment was acquired from Metrotec, S.A (Barcelona, Spain). The machine has a 5 kN load cell and is operated at a 2 mm/min speed. The tensile properties are the mean experimental values were obtained from 5 specimens for any of the composite formulations.

### 2.4. Specimen 3D Model and Injection Molding Simulation

A 3D model of the tensile specimen was obtained with the CAD software SolidWorks^®^ by Dassault Systemes (Aachen, Germany). The model was parametrized using the measures defined by ASTM D638. 

The injection molding of the specimen was simulated using the simulation add-on SolidWorks^®^ Plastics which simulated the composite flow inside the mold and the speed of the injection front. The injection molding gate was situated in the base of the specimen, and the rest of the injection parameters used to obtain the physical specimens were used in the simulation.

### 2.5. Morphological Analysis of the Reinforcements

The morphology of the fibers was analyzed with a MorFI Compact morphological analyzer by Techpap SAS (Gières, France). The fibers are introduced in an aqueous solution in the equipment that tests the length and width of 30,000 fibers. The equipment evaluates the mean and weighted length and the diameter of the fibers. The fibers are extracted from the matrix by solubilization of small pieces of composite in a Soxhlet apparatus. The solvent is decahydronaphthalene. The extraction process is operated for 24 h. 

## 3. Results and Discussion

### 3.1. Experimental Results

The BioPE matrix used to obtain the composites has a tensile strength of 18.41 ± 0.07 MPa, a Young’s Modulus of 1.13 ± 0.05 Gpa, and a strain at the maximum strength of 8.61 ± 0.15%. Tensile strength ($\sigma_{t}^{C}$) and strain at the break ($\varepsilon_{t}^{C}$) of abaca-fiber-reinforced BioPE composites were impacted by the percentage of reinforcement and by the strength of the interface between the reinforcement and the matrix (Table 1). In the table, *V^F^* is the reinforcement volume fraction and $E_{t}^{C}$ is the Young’s modulus of the composites. In the manuscript, the authors use superscript m to refer to the properties of the matrix, F for the reinforcement, and C for the composite. Mechanical properties show subscript t to signal that they are referring to tensile properties. 

On the one hand, the use of a coupling agent increased noticeably the tensile strength of the composites reinforced with 30 wt.% of AF. Composites without coupling agents increased the tensile strength of the matrix by 18%. This is an indication of the strengthening capabilities of abaca fibers as a BioPE reinforcement. Due to the different nature of polyolefin and natural fibers, the former being hydrophobic and the latter hydrophilic, composite materials mixing these phases usually show poor interfaces that do not provide good transmission of shear forces [55]. As a result, some natural-fiber-reinforced polyolefins show decreases in their tensile strength against fiber contents [56]. To address this issue, the use of compatibilizers that increase the chemical reactivity of the fiber surface can be used to increase the strength of the interface. In the case of abaca-fiber-reinforced BioPE, the effect of the coupling agent is noticeable (Table 1). The tensile strength of the composites increased noticeably with the percentage of coupling agent, showing a local maximum for an 8 wt.% MAPE content. In this case, the composite increased the tensile strength of the matrix by 45.6%. Thus, it might be assumed that the main reason for such an increase is the strengthening of the interface, evolving from a weak to a strong interface. The differences between the strengths of the composites with MAPE contents, from 0 to 8 wt.%, are statistically relevant as found from the ANOVA analysis. Table 1 shows that the effect of the coupling agent is neither constant nor linear, and increasing the percentage of MAPE from 8 to 10 wt.% resulted in a relative decrease in the tensile strength of the composite. The effect of 6 and 10 wt.% of MAPE on tensile strength is statistically similar. This can be caused by self-entanglement of the MAPE, decreasing the relative capacity of MAPE to create chemical bonds with the fiber surface [57]. The experimental data were used to establish an 8 wt.% MAPE content as the percentage of the coupling agent to use to obtain strong interfaces and thus maximize the tensile strength of the obtained composites. The impact of abaca fiber content can be observed in Table 1. The tensile strength of the composites at a 20, 40, and 50 wt.% abaca fiber content with an 8 wt.% MAPE increased with the reinforcement content. Compared with the matrix, these composites increased by 30.8%, 56.7%, and 61.4% of their tensile strength, respectively. The tensile strength of the composites with abaca fiber contents in the range from 20 to 40 wt.% evolved linearly against fiber volume fractions. The composite with a 50 wt.% abaca fiber content deviated from this linear behavior and returned values slightly lower than those previewed by a linear evolution. The percentage of the coupling agent is linked to the abaca fiber content, and thus increases with such content. Then, composites with 50 wt.% abaca contents could require less MAPE to optimize their contribution to obtain a strong interface while preventing self-interactions. The literature shows that the content of the coupling agent can be decreased when increasing the reinforcement contents to maximize the tensile strength of such materials [54]. Thus, after analyzing the results, it seems that the strength of the interface increased due to the use of the coupling agent, a strong interface was obtained by adding 8 wt.% of MAPE to the composites, and, finally, that the contribution of the reinforcements was not constant and decreased when its content increased.

Young’s modulus only changed slightly with MAPE content, and the evolution of such modulus against such content did not follow any pattern. The literature agrees on the residual impact of the strength of the interface over Young’s modulus of the composites in some cases [58,59]. On the other hand, Young’s modulus changed noticeably with abaca fiber contents and evolved linearly with the volume fraction of reinforcement. Regarding the composites that added an 8 wt.% MAPE, their Young’s moduli increased by 2.88, 3.33, 4.48, and 5.70 times compared to that of the matrix. After analyzing the results, in the case of abaca fibers as a BioPE reinforcement, the strength of the interface showed little impact over Young’s moduli of the composite materials. 

Regarding the strain at the break of the materials, it is impacted by the presence and dosage of the coupling agent and by the percentage of reinforcement. The mechanism affecting both factors is different. Adding a more fragile phase to a composite increases its brittleness and decreases the amount of deformation that the material can sustain without breaking. BioPE breaks under ductile fracture before an extensive deformation and the creation of a neck in the specimen. Therefore, the authors use the strain at maximum strength to characterize the material. As soon as abaca fibers are added to the composites, the breaking mechanism changes to brittle fracture characterized by crack creation and growth [60]. The increase in the strain at the break when the coupling agent percentage increases occurs due to the definition of Young’s modulus as the ratio between the tensile strength and the strain. Then, if Young’s modulus remains similar and the tensile strength increases, the breaking strain must increase to maintain the ratio. Meanwhile, the addition of reinforcements to a composite has a direct impact on its tensile strength, and Young’s modulus negatively impacts its capability to deform without breaking. In this case, the presence of a coupling agent increases the capabilities to deform without breaking and expands the usability of such materials. ANOVA analysis of the results shows that the impact of MAPE over the strain at the break differentiates between MAPE contents from 0 to 4 wt.% and 6 to 10 wt.%. In these MAPE content ranges, the impact of the coupling agent over the strain at the break cannot be considered statistically relevant. Another aspect affected by the maximum deformation of the composite materials is the contribution of the matrix to the strength of the composite. The higher the deformation, the higher the contribution of the matrix and the exploitation of its strengthening capabilities.

The main competitors of natural-fiber-reinforced biopolymers are glass-fiber-reinforced polyolefins. Table 2 shows the tensile properties of HDPE reinforced with glass fiber. The HDPE used for the composites exhibited a tensile strength of 14.1 ± 0.4 MPa, Young’s modulus of 0.85 ± 0.11 GPa, and a strain at a maximum strength of 11.1 ± 0.3%.

The results show a high impact of GF contents on the tensile properties of the composites. Only 10 wt.% of GF increased 66.9% the tensile strength of the matrix, and with a 30 wt.% GF content, the increase was 195.7%. In the case of Young’s modulus, the increases were 91.8%. 227.1% and 382.3% for the composites adding 10, 20, and 30 wt.% GF. Thus, GF shows high strengthening and stiffening capabilities, and at a low content can increase noticeably the mechanical properties of the matrix. Nonetheless, it is difficult to prepare GF-based composites with contents superior to 30 wt.%, because due to the attrition phenomena during composite preparation, GF tends to decrease its length, and this phenomenon increases with GF content.

**Figure 1:**
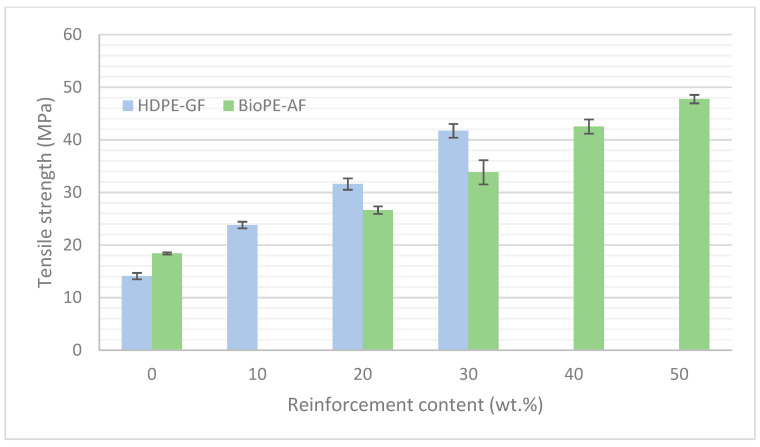
Comparison between the tensile strengths of AF-reinforced BioPE and GF-reinforced HDPE regarding reinforcement contents.

It can be seen that GF is more efficient as a strengthening constituent than abaca fibers. To obtain similar tensile strengths, the wt.% of AF must be 10% superior to that of GF (Figure 1). In any case, this can be seen as an advantage for AF-reinforced composites. On the one hand, AF is much cheaper than BioPE, and increasing the size of this phase allows for cheapening the BioPE-based composites and cutting back the economic advantage of HDPE compared to BioPE. On the other hand, the results show that AF-reinforced BioPE composites can reach mechanical competitiveness compared to GF-reinforced HDPE. In the case of Young’s modulus, the comparative is very similar to tensile strength, and while GF-based composites at the same reinforcement contents show higher Young’s moduli, AF-reinforced BioPE reaches similar Young’s modulus when the reinforcement wt.% is increased by 10%. Tensile properties of semi-oriented short-fiber-reinforced composites are impacted by the percentages of the phases and their intrinsic properties, the nature of these phases and thus their compatibility, the aspect ratio of the reinforcements, the relative orientation of the fibers against the loads, and the strength of the interface. All the composites are made in the same mold and equipment, and thus similar mean orientations are expected. Regarding the length of the fibers, such property is much more prone to change than the diameter due to fiber shortening during composite mixing. Data regarding the variation of such length are added in the next section. One of the main differences between natural fibers and glass fibers is the difficulty to obtain strong interfaces with polyolefin. Thus, to evaluate the weight of the strength of the interface on the final tensile properties of the composite, the authors use several micromechanical models to determine the contribution of the phases. 

### 3.2. Micromechanical Modeling

The first model that was used to evaluate the contribution of the phases was a modified rule of mixtures [51]:

(2) $$\sigma_{t}^{C}=f_{c}\sigma_{t}^{F}V^{F}+\left(1-V^{F}\right)\sigma_{t}^{m*}.$$

In the equation, the composite’s tensile strength, the fiber’s intrinsic tensile strength, and the matrix’s contribution are identified with $\sigma_{t}^{C}$, $\sigma_{t}^{F}$, and $\sigma_{t}^{m*}$, respectively. The volume fraction of reinforcement is annotated as $V^{F}$, and the volume fraction of the matrix is supposed to be $\left(1-V^{F}\right)$, assuming no porosity in the composite. A coupling factor ($f_{c}$) is introduced in the equation to take into account the strength of the interface, and the impact of the mean length and orientation of the reinforcements concerning the loads. This coupling factor does not appear in some rules of mixtures but is used in the case of aligned fibers. This can be misinterpreted as the mean orientation of the fibers having the highest impact on the contribution of such fibers on the tensile strength of the composite. While the mean orientation has a huge impact on the tensile properties of a semi-aligned short-fiber-reinforced composite, the role of the morphology of the reinforcements and the strength of the interface cannot be discarded. The coupling factor can be obtained by multiplying an orientation factor ($\chi_{1}$) by a length and interface factor ($\chi_{2}$), $f_{c}=\chi_{1}\chi_{2}$ [51]. The use of Equation (2) to preview the tensile strength of a composite has some limitations. On the one hand, the contribution of the matrix ($\sigma_{t}^{m*}$) is linked to the strain at the break of the composite, because it is the strain of the matrix in its stress–strain curve, corresponding to the strain at the break of the composite. Some authors suggest using the tensile strength of the matrix, assuming that the obtained tensile strength of the composite will be overestimated [51]. On the other hand, the contribution of the fibers is $f_{c}\sigma_{t}^{F},$ and this implies knowing two variables. The intrinsic tensile strength of the fibers can be measured by a single fiber tensile test, but the fibers can change their structure when they are mold-injected with the matrix and its tensile properties can change. As an example, some natural fibers have a lumen that collapse during composite preparation and specimen mold injection. Additionally, natural fibers tend to show a large scatter on their mechanical properties. Moreover, some authors accept that there are significant changes between mechanical properties measured from the fibers and the same properties calculated by using micromechanical models [61]. Moreover, the coupling factor allows estimating the strength of the interface, and the literature shows that semi-aligned short-fiber-reinforced composites with strong interfaces exhibit coupling factors ranging from 0.18 to 0.20 [62]. Therefore, the experimental values obtained for the tensile properties (Table 1) can be used to obtain the contribution of the reinforcement understood as $f_{c}\sigma_{t}^{F}$. Afterward, coupling factors ranging from 0.18 to 0.20 can be hypothesized for the coupled composites that show the highest tensile strengths to back-calculate a theoretical intrinsic tensile strength for the abaca fibers. Table 3 shows the contribution of the fibers, the theoretical intrinsic tensile strength of abaca fibers for the composites that added 8 wt.% of MAPE, the contribution of the matrix, and the theoretical coupling factor belonging to all the composite materials.

The contribution of the matrix was obtained from its stress–strain curve. To be able to obtain the values, the curve was fitted to the following equation:

(3) $$\sigma_{t}^{M*}={0.0007\left(\varepsilon_{t}^{C}\right)}^{5}-0.0238{\left(\varepsilon_{t}^{C}\right)}^{4}+{0.3066\left(\varepsilon_{t}^{C}\right)}^{3}-2.1009{\left(\varepsilon_{t}^{C}\right)}^{2}+8.5519\varepsilon_{t}^{C}+0.2685.$$

The contribution of the abaca fibers to the tensile strength of the composite was increasing with the percentage of MAPE until 8 wt.% was reached, then decreased. This is an indication of the increase in the strength of the interface and good exploitation of the strengthening capabilities of the fibers. The value also increased when the percentage of reinforcement increased up to 40 wt.%; then, there was a slight decrease in the composite reinforced with 50 wt.% of AF. This can be explained by the increasing difficulty to achieve a good dispersion of the fibers when their percentage increases.

The intrinsic tensile strength of AF obtained with the experimental values, Equation (2), and supposing coupling factors ranging from 0.18 to 0.20 showed a positive correlation with reinforcement contents in the range from 20 to 40 wt.% (Table 3). This increase in the tensile strength of the fibers is to be expected, because when it increases, the energy needed to prepare the composite increases, and the fibers tend to decrease in length. Then, purely by a decreasing the probability of finding a defect in the fiber when this fiber becomes shorter, its strength increases. Moreover, the obtained intrinsic tensile strengths of abaca fibers are inside the values reported in the literature [13,14]. The theoretical intrinsic strength of the fibers decreased for the composite with 50 wt.% of AF, indicating that its coupling factor is lower than that of the other composites.

The theoretical coupling factors for the composites at different MAPE contents were evaluated using the intrinsic tensile strength found for the composite at 8 wt.% MAPE. These coupling factors showed a positive correlation with the percentage of MAPE up to 8 wt.%; then, a slight decrease was observed. Since the constituents of the composite are the same for the 30 wt.% AF-reinforced composites, it can be assumed that the changes can be attributed to the increase in the strength of the interface because the morphology and mean orientation of the fibers are expected to be similar.

**Figure 2:**
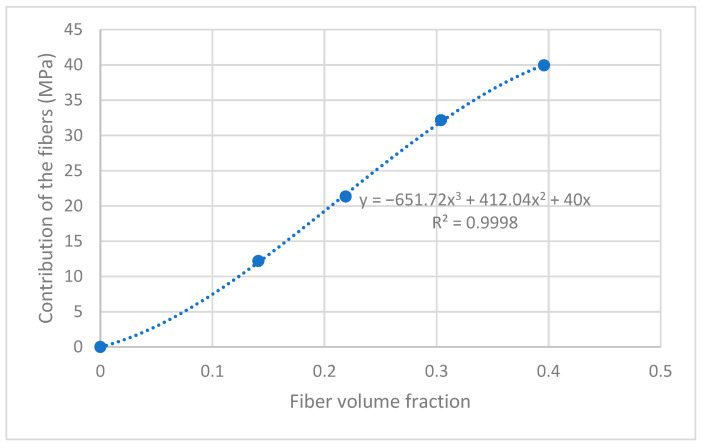
Contribution of the fibers to the tensile strength of the composite against fiber volume fraction.

Figure 2 shows the evolution of the contribution of the fibers against their volume fraction.

The contribution of the fibers is obtained from Equation (2) as $\sigma_{t}^{C}-\left(1-V^{F}\right)\sigma_{t}^{m*}=f_{c}\sigma_{t}^{F}V^{F}$ to evaluate the contribution as a function of the fiber volume fraction. The contribution increases with fiber volume fraction almost linearly. Nonetheless, the best fit is found with an S-curve. This curve indicates, on the one hand, the possible existence of a critical fiber volume fraction [63]. Below this critical volume fraction, the strength of the composite is expected to decrease concerning the matrix. Then, the curve shows a quasi-linear zone for AF percentages in the range of 20 to 40 wt.%. Higher fiber volume fractions decrease the contribution of the reinforcements to the tensile strength of the matrix. This can be caused by a non-optimal dispersion of the reinforcements, an incorrect dosage of a coupling agent resulting in self-entanglements of such coupling agent, or the existence of a superior critical fiber volume fraction that limits the functional amount of reinforcement. 

The modified rule of mixtures was used to obtain an indication of the strength of the interface and its evolution against coupling agent and reinforcement contents. Nonetheless, the equation showed two unknowns, the coupling factor and the intrinsic tensile strength of abaca fibers. The authors used more refined micromechanical models to validate their findings.

Based on a Cox shear lag model that uses the length and interface factor ($\chi_{2}$), the literature shows that such a factor can be obtained from the following equation [51]:

(4) $$\chi_{2}=1-\frac{tanh\left(\frac{\beta xL_{F}}{2}\right)}{\frac{\beta xL_{F}}{2}}.$$

In the equation, $L_{F}$ is the mean length of the fibers (Table 4) and β can be obtained with the following equation:

(5) $$\beta =\frac{2}{D_{F}}\sqrt{\frac{2G_{M}}{E_{t}^{F}xln\left(\frac{P_{F}}{V^{F}}\right)}},$$

where $P_{F}$ is a fiber packing factor that equals pi for square packing and $D_{F}$ the mean diameter of the fiber, in this case, 19.5 μm. $G_{M}$ is the shear modulus of the matrix, approximated using the following equation:

(6) $$G_{M}=\frac{E_{t}^{M}}{2\left(\nu +1\right)}.$$

In the equation, ν is the Poisson’s ratio of the matrix with a value of 0.45 [64].

The intrinsic Young’s modulus of the matrix was obtained with Hirsch’s model in previous research, with a mean value of 33.1 GPa.

After using the mentioned equations, the length and interface factors for the fibers with AF contents are shown in Table 4. In the table, $f_{c}^{1}$, $f_{c}^{2}$, and $f_{c}^{3}$ correspond to 0.25, 0.3, and 0.35 values for $\chi_{1}$.

The values obtained for the length and interface factor with a mean value of 0.603 are in line with the values that can be found in the literature for other natural-fiber-reinforced polyolefins. Thus, the obtained values are accepted as plausible [62]. 

**Figure 3:**
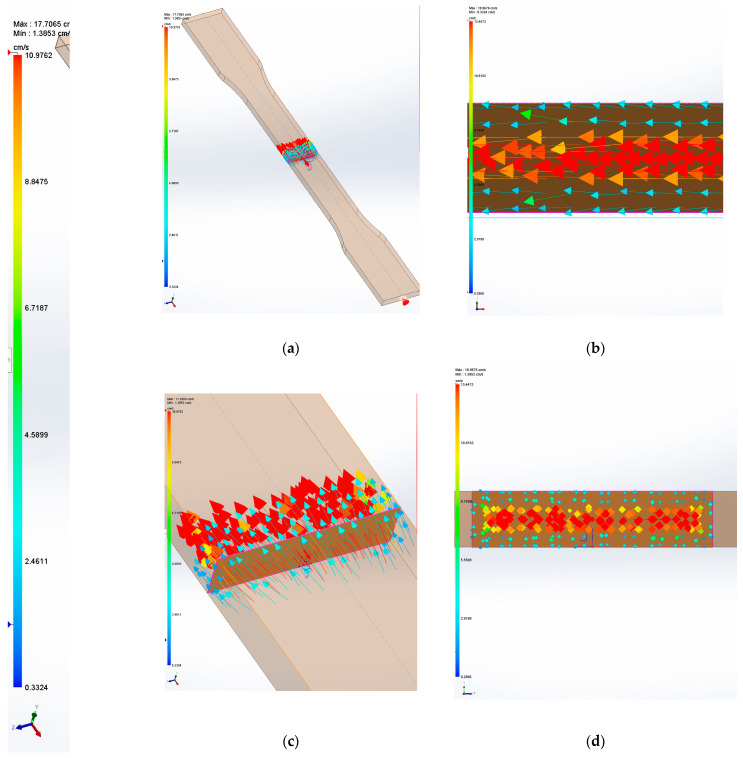
Simulation of the speed of the polymer during mold injection: (**a**) Isometric view of a dog bone specimen with the position of the injection point; (**b**) Longitudinal section of the specimen; (**c**) Isometric detail of a transversal section; (**d**) Detail of the transversal section.

Short-fiber-reinforced composites, when mold-injected, show a non-random orientation. These materials show three orientation zones: the skin, the shell, and the core, where the skin is the zone in contact with the mold and the core is the central region [65]. The shell is the transition area between the skin and the shell. When a composite is mold-injected, its velocity at the skin is lower than that in the core due to the friction phenomena that occur in this zone. At the same time, the fibers tend to align in the skin with the direction of the shear loads that coincide with the direction of the advancing composite. In the case of a standard specimen, the fiber is aligned with the tensile loads. In the core region, the fibers tend to show a more random orientation. The shell region shows a semi-orientation as it is the interface between the skin and core regions [66,67]. Figure 3 shows the speed of a BioPE when it is mold-injected in the shape of a dog bone specimen. The direction of the fluid is marked by the vectors. The color of each vector indicates the forward speed, according to the color code of the same figure. 

It can be observed that the velocity of the composite in contact with the mold (blue arrows) is lower than the speed of the core region (red arrows) with a rate of 4.6, which is in line with the literature for highly filled composites with a rate of 4.5 [68]. The resulting semi-orientation of the fibers directly impacts the properties of the composite. The higher the orientation, the higher the contribution of the fibers. Random-oriented composites have orientation angles of around 0.2 [69]. The literature shows that dog bone specimens obtained by mold-injecting short-fiber-reinforced composites show a semi-orientation due to the alignment of the fibers in the skin and shell region and tend to return orientation factors in the range from 0.25 to 0.35 [53]. The higher the value, the thicker the skin and shell regions. The thickness of these regions is known to be positively correlated with the length of the fiber and negatively correlated with the percentage of fibers [67]. Table 4 shows the coupling factors corresponding to the calculated length and interface factors and orientation factors with values of 0.25, 0.30, and 0.35. 

The coupling factors obtained with an orientation factor equal to 0.30 with an average value of 0.18 agree with the values obtained from the modified rule of mixtures and an expected strong interface. The other values returned coupling factors lower or higher than expected. This second approach corroborates the strength of the obtained interface.

Another model that can be used is the Kelly and Tyson equation:

(7) $$\sigma_{t}^{C}=\chi_{1}\left[\sum_{l=0}^{l=lc}\left[\frac{\tau \cdot l\cdot V_{l}^{F}}{D_{F}}\right]+\sum_{l=lc}^{\infty }\left[\sigma_{t}^{F}\cdot V_{l}^{F}\cdot \left(1-\frac{\sigma_{t}^{F}\cdot D_{F}}{4\cdot \tau \cdot l}\right)\right]\right]+\left(1-V^{F}\right)\cdot \sigma_{t}^{m*}.$$

In the equation, τ in the interfacial shear strength that evaluates the ability of the interface to convey loads from the matrix to the fiber surface. The highest load coincides with the shear strength of the matrix. The length of the fibers is evaluated differently depending on whether such fiber is subcritical or supercritical. For a fiber to be supercritical, its length must be sufficient to accumulate the loads that equal its tensile strength and then break. Thus, the supercritical fiber failure mechanism is breakage. Subcritical fibers do not break as their length does not allow accumulating enough loads, and their failure mechanism is slippage or fiber pull-out [70,71].

The critical length is defined by the following equation:

(8) $$l_{C}=\frac{\sigma_{t}^{F}D_{F}}{2\tau }.$$

The stronger the fiber, the longer the critical length, and the stronger the interface, the shorter the critical length. The diameter increases the area of the section of the fiber as well as the area of the surface of the fiber where the shear loads are transmitted from the matrix to the fiber. Nonetheless, the area of the section is related to the square of the diameter while the area of the fiber surface is determined by the diameter. Thus, the larger the diameter, the higher the critical length. Then, Equation (7) determined the contributions of subcritical fibers ($X^{\prime }$), supercritical fibers ($Y^{\prime }$), and the matrix (Z); it can be written as $\sigma_{t}^{C}=\chi_{1}\left(X^{\prime }+Y^{\prime }\right)+Z$.

In its original shape, Kelly and Tyson equation shows three unknowns, the orientation factor ($\chi_{1}$), the interfacial strength (τ), and the intrinsic tensile strength of the reinforcements ($\sigma_{t}^{F}$). Anyhow, Bowyer and Bader proposed a method to solve the Kelly and Tyson model. This solution is based on assuming that all the phases are exposed to the same deformations, that σ=Eε, and thus that the contribution of the fiber at any strain is $\sigma_{t}^{F}=E_{t}^{F}\varepsilon_{t}^{F}=E_{t}^{F}\varepsilon_{t}^{C}$. Then, the proposed solution uses two strain points between 0 and the strain at the break of the composite and allows computing the orientation factor and the interfacial shear strength using numerical methods such as the bisection method.

**Figure 4:**
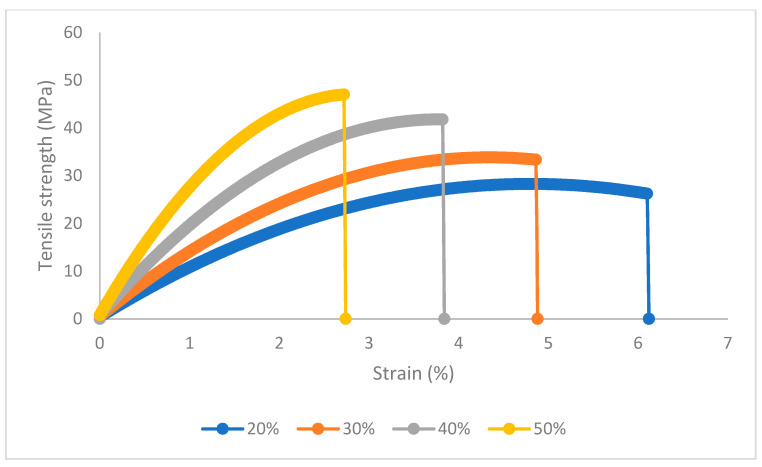
Stress–strain curves of the BioPE-based composite materials adding 20 to 50 wt.% AF and 8 wt.% MAPE.

Bowyer and Bader’s solution assumptions are difficult to accomplish with natural-fiber-reinforced composites because the elastic regions of their stress–strain curves are difficult to identify. Moreover, the solution is highly sensitive to small changes in the experimental values and can return a solution, but this solution can contradict the literature. In this case, the authors obtained the solutions shown in Table 5. Figure 4 shows the stress–strain curves of the composite materials.

While the orientation factors keep inside the 0.25 to 0.35 expected range (mean orientation angles (α) from 45.0° to 39.7°, taking into account that $\chi_{1}={cos}^{4}(\alpha )$), the rest of the micromechanical parameters are not expected. Nonetheless, a more homogeneous orientation factor is to be anticipated, because the variations in length (Table 4) are not so significant. Moreover, the larger the fibers, the more oriented they are expected to be; the orientation factor does not fulfill this condition and increases for the shortest fibers. On the one hand, the interfacial shear strengths are lower than expected and are far from the previewed 10.42 MPa with von Mises Criteria ($\tau =\sigma_{t}^{M}/\sqrt{3}$), except the composite with 40 wt.% of AF [71,72]. Moreover, the intrinsic tensile strengths for AF obtained with the Kelly and Tyson equation from the orientation factors and interfacial tensile strengths computed by Bowyer and Bader’s solution are high and out of the range available in the literature. Thus, the solutions provided by Bowyer and Bader were discarded. The authors solved the Kelly and Tyson equation by formulating the following hypothesis: (i) the orientation factor is 0.30 (42.3°), the value found in the literature and in the present research, and (ii) the interface is strong and in the vicinity of an optimal value, and thus the interfacial strength is obtained with von Mises Criteria.

The obtained intrinsic tensile strength is shown in Table 6.

**Figure 5:**
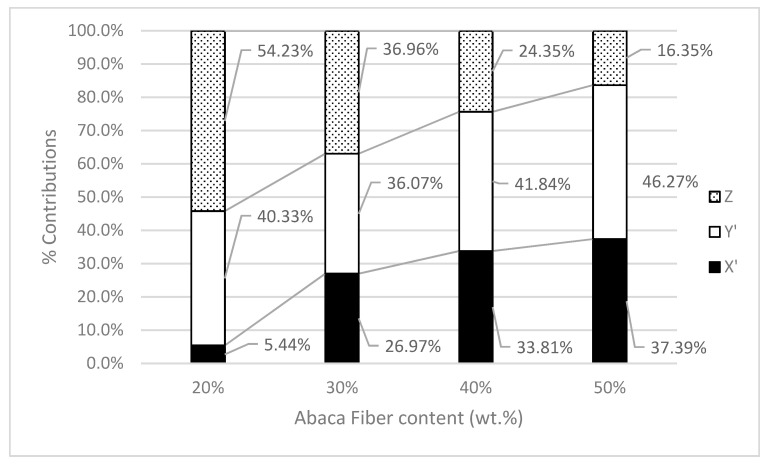
Contribution of the matrix (Z), the subcritical (X′) and supercritical (Y′) fibers to the tensile strength of the composite against fiber volume fraction.

The values for the intrinsic tensile strengths are inside the experimental values found in the literature, which makes them plausible. The intrinsic tensile strengths obtained with the Kelly and Tyson equation are different from the ones obtained with the modified rule of mixtures. The main difference between the two methods is that the modified rule of mixtures considers that all the fibers are equal and contribute the same to the tensile strength of the composite. The Kelly and Tyson equation distinguishes between subcritical and supercritical fibers since their contributions are distinct. Moreover, the Kelly and Tysos equation can be used from single fiber to single fiber if the data are available, or employing percentages as performed in the present work. Figure 5 shows the contributions in the percentage of the subcritical and supercritical fibers as well as the matrix to the tensile strength of the composites.

The contribution of the matrix is negatively correlated with the increase in reinforcement. This is expected because the percentage of the matrix decreases and the strain at the break of the composite does the same. These two circumstances limit the contribution of the matrix.

The contribution of AF fibers increases from 45.8% to 86.7%, as expected due to a larger presence of this reinforcement. Nonetheless, while the contribution of supercritical fibers remains stable, in the range from 36.1% to 41.8%, the contribution of subcritical fibers increases from 5.4% to 37.4%. Thus, the mix of the contribution is not the same for all the composites. This is the main difference between the modified rule of mixtures and the Kelly and Tyson equation when the contribution of the reinforcement is evaluated, and one of the main reasons why the coupling factor computed from the intrinsic tensile strengths in Table 4 tends to decrease. These coupling agents oppose the interfacial shear strength because this parameter indicates a strong interface and the coupling factor indicates a weak one. Thus, when using micromechanical parameters obtained with some model in another model, the assumptions made with both models must be taken into account to prevent misinterpretations of the results.

The assayed models allow us to affirm that the BioPE composites reinforced with AF showed strong interfaces for 20 to 40 wt.% AF contents and a good exploitation of the strengthening capabilities of AF. The composite at a 50 wt.% AF content showed a weaker interface and lower exploitation of the strengthening capabilities of AF. This can be attributed to a MAPE dosage higher than the optimal or to an improvable dispersion of the reinforcing fibers.

## 4. Conclusions

The growing environmental awareness increases the importance of research on materials with enhanced environmental impact. This includes the use of renewable resources and the design of recyclable and sustainable materials. Thus, it isimportant to find new industry-friendly materials that exhibit mechanical and transformation properties similar to commodities but with a lower environmental impact.

The present work focuses on the research of biocomposite materials based on a BioPE reinforced with abaca fibers. The use of a BioPE, with chemical and mechanical properties similar or equal to HDPE, can eliminate some market entry barriers for the industry. Nonetheless, its cost, higher than that of HDPE, can be a drawback. Here, the use of abaca fibers has two missions. On the one hand, these fibers acting as reinforcement allows obtaining composite materials with mechanical properties similar to those of glass-fiber-reinforced HDPE. On the other hand, the use of a reinforcement that is cheaper than the matrix reduces the cost of the composites. The authors have successfully mold-injected a composite, adding 50 wt.% of abaca fibers, occupying 39.6% of the volume of the composite and reaching mechanical properties higher than those of glass fiber composites at a 30 wt.% content. These bio-based materials can be of the interest for automotive and product design sectors.

The use of micromechanical models is a way to determine the contribution of the phases to the properties of a composite. All micromechanical models have a set of assumptions and limitations that must be determined to accept their results. In this work, the strength of the interface and the intrinsic tensile strength of abaca fibers were explored with four different approaches. The modified rule of mixtures, the length and interface factor, and the Kelly and Tyson equation returned sensible results. On the other hand, the use of Bowyer and Bader’s solution to the Kelly and Tyson equation while providing a solution delivered values in contradiction with the literature. The models stated that the interface between abaca fibers and BioPE was strong when an 8 wt.% of coupling agent was added to the composites.

This paper shows the ability of fully bio-based composites to compete with oil-based composite commodities. Moreover, a bio-based high-density polyethylene with chemical and mechanical properties similar or equal to commercial oil-based HDPE can overcome some industry entry barriers that complicate the application of other, less known bio-based polymers. Moreover, adding a natural-fiber reinforcement helps enhance the mechanical properties to the level of GF-reinforced polyolefin and diminishes the cost of the bio-based polymer.

## Figures and Tables

**Table 1:** Tensile strength, Young’s modulus, and strain at the break of abaca-fiber-reinforced BioPE composites against reinforcement content and coupling agent dosage.

AF Content (%)	MAPE Content (%)	*V^F^*	$\sigma_{t}^{C}$ (MPa)	$E_{t}^{C}$ (GPa)	$\varepsilon_{t}^{C}$ (%)
20	8	0.141	26.64 ± 0.24 ^a^	3.25 ± 0.03 ^a^	6.10 ± 0.29 ^a^
30	0	0.219	22.46 ± 0.54 ^b^	3.47 ± 0.02 ^b^	3.18 ± 0.25 ^bc^
	2		24.97 ± 0.26 ^c^	3.50 ± 0.06 ^bc^	3.24 ± 0.26 ^bc^
	4		28.56 ± 0.70 ^d^	3.28 ± 0.02 ^cd^	3.72 ± 0.27 ^cd^
	6		31.52 ± 0.23 ^ef^	3.74 ± 0.01 ^ef^	4.53 ± 0.30 ^de^
	8		33.85 ± 0.77 ^h^	3.76 ± 0.04 ^f^	4.86 ± 0.20 ^e^
	10		32.56 ± 0.36 ^fg^	3.64 ± 0.04 ^ce^	4.96 ± 0.21 ^e^
40	8	0.304	42.51 ± 0.45 ^i^	5.06 ± 0.01 ^g^	3.82 ± 0.26 ^f^
50	8	0.396	47.73 ± 0.27 ^j^	6.44 ± 0.11 ^h^	2.70 ± 0.13 ^g^

Different letters a, b, c, d, e, f, g, h, i, and j represent the statistical difference (ANOVA, *p* < 0.05) between the properties of the materials.

**Table 2:** Tensile strength, Young’s modulus, and strain at the break of glass-fiber-reinforced HDPE composites against reinforcement content.

GF Content (%)	V^F^	$\sigma_{t}^{C}$ (MPa)	$E_{t}^{C}$ (GPa)	$\varepsilon_{t}^{C}$ (%)
10	0.041	23.8 ± 0.21	1.63 ± 0.01	5.1 ± 0.14
20	0.088	31.6 ± 0.36	2.78 ± 0.01	4.6 ± 0.08
30	0.143	41.7 ± 0.44	4.10 ± 0.01	4.3 ± 0.12

**Table 3:** Parameters used to model the micromechanics of BioPE composites reinforced with AF.

AF Content (wt.%)	MAPE Content (wt.%)	$\sigma_{t}^{m*}$ (MPa)	$f_{c}\sigma_{t}^{F}$ (MPa)	$\sigma_{t}^{F\;(1)}$ (MPa)	$f_{c}^{}$
20	8	16.81	86.53	480.7–432.6	0.18–0.20
30	0	15.12	48.64		0.09–0.10
	2	15.21	59.78		0.11–0.12
	4	15.87	73.82		0.14–0.15
	6	16.49	85.12		0.16–0.17
	8	16.02	97.44	541.3–4872	0.18–0.20
	10	16.11	91.22		0.17–0.19
40	8	14.87	105.79	587.7–528.9	0.18–0.20
50	8	12.91	100.84	560.2–504.2	0.18–0.20

^(1)^ The values were obtained from Equation (1) with coupling factor values between 0.18 and 0.20.

**Table 4:** Length and interface factors obtained for the composites adding 8 wt.% of coupling agent.

AF Content (wt.%)	$\chi_{2}$	$L_{F}^{}$ (µm)	$f_{c}^{}$ ^1^	$f_{c}^{}$ ^2^	$f_{c}^{}$ ^3^
20	0.598	567.6	0.150	0.179	0.209
30	0.603	532.4	0.150	0.181	0.211
40	0.603	498.3	0.151	0.181	0.211
50	0.608	475.5	0.152	0.182	0.212

^1^ $\chi_{1}=0.25$, ^2^ $\chi_{1}=0.30$, ^3^ $\chi_{1}=0.35$.

**Table 5:** Experimental parameters were used to solve Kelly and Tyson equation with the solution proposed by Bowyer and Bader and obtained results.

AF Content (wt.%)	20%	30%	40%	50%
Strain level 1 analyzed (%)	2.01	1.60	1.27	0.89
Strain level 2 analyzed (%)	4.03	3.21	2.55	1.78
Composite stress at strain level 1 (MPa)	20.14	22.10	25.98	27.53
Composite stress at strain level 2 (MPa)	26.31	30.06	35.25	38.25
Matrix stress at strain level 1 (MPa)	11.10	9.69	8.32	6.42
Matrix stress at strain level 2 (MPa)	15.14	13.92	12.56	10.34
Orientation factor	0.29	0.31	0.25	0.35
Interfacial shear strength (MPa)	5.72	6.56	10.39	6.83
Critical length (µm)	1956	1835	1239	1501
Intrinsic tensile strength (MPa)	1148	1235	1321	1051

**Table 6:** Intrinsic tensile strength of AF obtained from Kelly and Tyson equation.

AF Content (wt.%)	20%	30%	40%	50%
Orientation factor	0.30	0.30	0.30	0.30
Interfacial shear strength (MPa)	10.42	10.42	10.42	10.42
Critical length (µm)	425.3	583.7	772.3	736.4
Intrinsic tensile strength (MPa)	454.5	623.8	825.3	787.0
fc	0.19	0.16	0.13	0.13

## Data Availability

Not applicable.

## References

[1] Das S.C., La Rosa A.D., Grammatikos S.A. (2022). Life cycle assessment of plant fibers and their composites. Plant Fibers, their Composites, and Applications.

[2] Walker S., Rothman R. (2020). Life cycle assessment of bio-based and fossil-based plastic: A review. J. Clean. Prod..

[3] Spierling S., Röttger C., Venkatachalam V., Mudersbach M., Herrmann C., Endres H.-J. (2018). Bio-based Plastics—A Building Block for the Circular Economy?. Procedia CIRP.

[4] Milani A.S., Eskicioglu C., Robles K., Bujun K., Hosseini-Nasab H. (2011). Multiple criteria decision making with life cycle assessment for material selection of composites. Express Polym. Lett..

[5] Rebitzer G., Ekvall T., Frischknecht R., Hunkeler D., Norris G., Rydberg T., Schmidt W.P., Suh S., Weidema B.P., Pennington D.W. (2004). Life cycle assessment Part 1: Framework, goal and scope definition, inventory analysis, and applications. Environ. Int..

[6] Pandey J., Nagarajan V., Mohanty A., Misra M. (2015). Commercial potential and competitiveness of natural fiber composites. Biocomposites.

[7] Kumar S., Manna A., Dang R.J.M.T.P. (2022). A review on applications of natural Fiber-Reinforced composites (NFRCs). Mater. Today Proc..

[8] Lau K.-t., Hoi-yan Cheung K., Hui D. (2009). Natural fiber composites. Compos. Part B Eng..

[9] Ma X.F., Yu J.G., Kennedy J.F. (2005). Studies on the properties of natural fibers-reinforced thermoplastic starch composites. Carbohydr. Polym..

[10] Malkapuram R., Kumar V., Negi Y.S. (2009). Recent Development in Natural Fiber Reinforced Polypropylene Composites. J. Reinf. Plast. Compos..

[11] Mohanty A.K., Misra M., Drzal L.T. (2002). Sustainable bio-composites from renewable resources: Opportunities and challenges in the green materials world. J. Polym. Environ..

[12] Ashby M.F., Cebon D. (1993). Materials selection in mechanical design. J. Phys..

[13] Sinha A.K., Bhattacharya S., Narang H.K. (2021). Abaca fibre reinforced polymer composites: A review. J. Mater. Sci..

[14] Delicano J.A. (2018). A review on abaca fiber reinforced composites. Compos. Interfaces.

[15] Granda L.A., Espinach F.X., Lopez F., Garcia J.C., Delgado-Aguilar M., Mutje P. (2016). Semichemical fibres of Leucaena collinsii reinforced polypropylene: Macromechanical and micromechanical analysis. Compos. Pt. B-Eng..

[16] Výbohová E., Kučerová V., Andor T., Balážová Ž., Veľková V. (2018). The effect of heat treatment on the chemical composition of ash wood. BioResources.

[17] Sain M., Suhara P., Law S., Bouilloux A. (2005). Interface modification and mechanical properties of natural fiber-polyolefin composite products. J. Reinf. Plast. Compos..

[18] Piggott M. (1987). The effect of the interface/interphase on fiber composite properties. Polym. Compos..

[19] Charlet K., Béakou A. (2011). Mechanical properties of interfaces within a flax bundle–Part I: Experimental analysis. Int. J. Adhes. Adhes..

[20] Asumani O.M.L., Reid R.G., Paskaramoorthy R. (2012). The effects of alkali-silane treatment on the tensile and flexural properties of short fibre non-woven kenaf reinforced polypropylene composites. Compos. Part A-Appl. Sci. Manuf..

[21] Bledzki A.K., Fink H.P., Specht K. (2004). Unidirectional hemp and flax EP- and PP-composites: Influence of defined fiber treatments. J. Appl. Polym. Sci..

[22] Cao Y., Shibata S., Fukumoto I. (2006). Mechanical properties of biodegradable composites reinforced with bagasse fibre before and after alkali treatments. Compos. Part A-Appl. Sci. Manuf..

[23] Colom X., Carrasco F., Pages P., Canavate J. (2003). Effects of different treatments on the interface of HDPE/lignocellulosic fiber composites. Compos. Sci. Technol..

[24] Karthi N., Kumaresan K., Sathish S., Gokulkumar S., Prabhu L., Vigneshkumar N. (2020). An overview: Natural fiber reinforced hybrid composites, chemical treatments and application areas. Mater. Today Proc..

[25] Oliver-Ortega H., Julian F., Espinach F.X., Tarrés Q., Ardanuy M., Mutjé P. (2019). Research on the use of lignocellulosic fibers reinforced bio-polyamide 11 with composites for automotive parts: Car door handle case study. J. Clean. Prod..

[26] Kumar R., Ul Haq M.I., Raina A., Anand A. (2019). Industrial applications of natural fibre-reinforced polymer composites—Challenges and opportunities. Int. J. Sustain. Eng..

[27] Delgado-Aguilar M., Tarres Q., Marques M.d.F.V., Espinach F.X., Julian F., Mutje P., Vilaseca F. (2019). Explorative Study on the Use of Curaua Reinforced Polypropylene Composites for the Automotive Industry. Materials.

[28] Averous L., Fringant C., Moro L. (2001). Starch-based biodegradable materials suitable for thermoforming packaging. Starch-Starke.

[29] Ferreira F.V., Pinheiro I.F., Mariano M., Cividanes L.S., Costa J.C., Nascimento N.R., Kimura S.P., Neto J.C., Lona L.M. (2019). Environmentally friendly polymer composites based on PBAT reinforced with natural fibers from the amazon forest. Polym. Compos..

[30] Balart J.F., Fombuena V., Fenollar O., Boronat T., Sánchez-Nacher L. (2016). Processing and characterization of high environmental efficiency composites based on PLA and hazelnut shell flour (HSF) with biobased plasticizers derived from epoxidized linseed oil (ELO). Compos. Part B Eng..

[31] Bodros E., Pillin I., Montrelay N., Baley C. (2007). Could biopolymers reinforced by randomly scattered flax fibre be used in structural applications?. Compos. Sci. Technol..

[32] Flores-Hernández C., Colín-Cruz A., Velasco-Santos C., Castaño V., Rivera-Armenta J., Almendarez-Camarillo A., García-Casillas P., Martínez-Hernández A. (2014). All Green Composites from Fully Renewable Biopolymers: Chitosan-Starch Reinforced with Keratin from Feathers. Polymers.

[33] Islam M.Z., Sarker M.E., Rahman M.M., Islam M.R., Ahmed A.F., Mahmud M.S., Syduzzaman M. (2022). Green composites from natural fibers and biopolymers: A review on processing, properties, and applications. J. Reinf. Plast. Compos..

[34] Siracusa V., Blanco I. (2020). Bio-polyethylene (Bio-PE), Bio-polypropylene (Bio-PP) and Bio-poly (ethylene terephthalate)(Bio-PET): Recent developments in bio-based polymers analogous to petroleum-derived ones for packaging and engineering applications. Polymers.

[35] Gowthaman N., Lim H., Sreeraj T., Amalraj A., Gopi S. (2021). Advantages of biopolymers over synthetic polymers: Social, economic, and environmental aspects. Biopolymers and Their Industrial Applications.

[36] Mendieta C.M., Vallejos M.E., Felissia F.E., Chinga-Carrasco G., Area M.C. (2020). Bio-polyethylene from wood wastes. J. Polym. Environ..

[37] Chauhan V., Kärki T., Varis J. (2022). Review of natural fiber-reinforced engineering plastic composites, their applications in the transportation sector and processing techniques. J. Thermoplast. Compos. Mater..

[38] Suriani M., Ilyas R., Zuhri M., Khalina A., Sultan M., Sapuan S., Ruzaidi C., Wan F.N., Zulkifli F., Harussani M. (2021). Critical review of natural fiber reinforced hybrid composites: Processing, properties, applications and cost. Polymers.

[39] Garcia-Garcia D., Carbonell-Verdu A., Jordá-Vilaplana A., Balart R., Garcia-Sanoguera D. (2016). Development and characterization of green composites from bio-based polyethylene and peanut shell. J. Appl. Polym. Sci..

[40] Bazan P., Nosal P., Kozub B., Kuciel S.J.M. (2020). Biobased polyethylene hybrid composites with natural fiber: Mechanical, thermal properties, and micromechanics. Mater. Today Proc..

[41] Bazan P., Mierzwiński D., Bogucki R., Kuciel S.J.M. (2020). Bio-based polyethylene composites with natural fiber: Mechanical, thermal, and ageing properties. Materials.

[42] Serra-Parareda F., Tarrés Q., Delgado-Aguilar M., Espinach F.X., Mutjé P., Vilaseca F.J.M. (2019). Biobased composites from biobased-polyethylene and barley thermomechanical fibers: Micromechanics of composites. Materials.

[43] Kelly A., Tyson W. (1965). Tensile porperties of fibre-reinforced metals—Copper/tungsten and copper/molybdenum. J. Mech. Phys. Solids.

[44] Aguado R., Espinach F.X., Vilaseca F., Tarrés Q., Mutjé P., Delgado-Aguilar M. (2022). Approaching a Zero-Waste Strategy in Rapeseed (*Brassica napus*) Exploitation: Sustainably Approaching Bio-Based Polyethylene Composites. Sustainability.

[45] Tarrés Q., Hernández-Díaz D., Ardanuy M. (2021). Interface Strength and Fiber Content Influence on Corn Stover Fibers Reinforced Bio-Polyethylene Composites Stiffness. Polymers.

[46] Barbalho G.H.d.A., Nascimento J.J.d.S., Silva L.B.d., Gomez R.S., Farias D.O.d., Diniz D.D.S., Santos R.S., Figueiredo M.J.d., Lima A.G.B.d. (2023). Bio-Polyethylene Composites Based on Sugar Cane and Curauá Fiber: An Experimental Study. Polymers.

[47] Seculi F., Espinach F.X., Julián F., Delgado-Aguilar M., Mutjé P., Tarrés Q. (2022). Evaluation of the Strength of the Interface for Abaca Fiber Reinforced Hdpe and Biope Composite Materials, and Its Influence over Tensile Properties. Polymers.

[48] Seculi F., Espinach F.X., Julián F., Delgado-Aguilar M., Mutjé P., Tarrés Q. (2023). Comparative Evaluation of the Stiffness of Abaca-Fiber-Reinforced Bio-Polyethylene and High Density Polyethylene Composites. Polymers.

[49] Thomason J.L. (2002). Interfacial strength in thermoplastic composites—At last an industry friendly measurement method?. Compos. Part A-Appl. Sci. Manuf..

[50] Tham M.W., Fazita M.N., Abdul Khalil H., Mahmud Zuhudi N.Z., Jaafar M., Rizal S., Haafiz M.M. (2019). Tensile properties prediction of natural fibre composites using rule of mixtures: A review. J. Reinf. Plast. Compos..

[51] Yan J., Demirci E., Gleadall A. (2023). Are classical fibre composite models appropriate for material extrusion additive manufacturing? A thorough evaluation of analytical models. Addit. Manuf..

[52] Bowyer W.H., Bader H.G. (1972). On the reinforcement of thermoplastics by imperfectly aligned discontinuous fibres. J. Mater. Sci..

[53] Reixach R., Franco-Marquès E., El Mansouri N.-E., de Cartagena F.R., Arbat G., Espinach F.X., Mutjé P. (2013). Micromechanics of Mechanical, Thermomechanical, and Chemi-Thermomechanical Pulp from Orange Tree Pruning as Polypropylene Reinforcement: A Comparative Study. Bioresources.

[54] Hernández-Díaz D., Villar-Ribera R., Julián F., Tarrés Q., Espinach F.X., Delgado-Aguilar M. (2020). Topography of the Interfacial Shear Strength and the Mean Intrinsic Tensile Strength of Hemp Fibers as a Reinforcement of Polypropylene. Materials.

[55] Zhou Y., Fan M., Chen L. (2016). Interface and bonding mechanisms of plant fibre composites: An overview. Compos. Part B Eng..

[56] Naghmouchi I., Mutjé P., Boufi S. (2015). Olive stones flour as reinforcement in polypropylene composites: A step forward in the valorization of the solid waste from the olive oil industry. Ind. Crops Prod..

[57] Mohanty S., Verma S.K., Nayak S.K. (2006). Dynamic mechanical and thermal properties of MAPE treated jute/HDPE composites. Compos. Sci. Technol..

[58] Fukuda H., Kawata K. (1974). On Young’s modulus of short fibre composites. Fibre Sci. Technol..

[59] Granda L.A., Espinach F.X., Mendez J.A., Tresserras J., Delgado-Aguilar M., Mutje P. (2016). Semichemical fibres of Leucaena collinsii reinforced polypropylene composites: Young’s modulus analysis and fibre diameter effect on the stiffness. Compos. Pt. B-Eng..

[60] Mouritz A. (2012). Fracture processes of aerospace materials. Introd. Aerosp. Mater..

[61] Shah D.U., Nag R.K., Clifford M.J. (2016). Why do we observe significant differences between measured and ‘back-calculated’ properties of natural fibres?. Cellulose.

[62] Reixach R., Espinach F.X., Franco-Marquès E., Ramirez de Cartagena F., Pellicer N., Tresserras J., Mutjé P. (2013). Modeling of the tensile moduli of mechanical, thermomechanical, and chemi-thermomechanical pulps from orange tree pruning. Polym. Compos..

[63] Maalej M., Hashida T., Li V.C. (1995). Effect of fiber volume fraction on the off-crack-plane fracture energy in strain-hardening engineered cementitious composites. J. Am. Ceram. Soc..

[64] Beijer J., Spoormaker J. (2000). Modelling of creep behaviour in injection-moulded HDPE. Polymer.

[65] Bayer R.J. (2013). Valoración de Materiales Compuestos de HDPE Reforzados con Fibras de Agave Sisalana. Aproximación a un Paradigma de Geometría Fractal para las Fibras. Ph.D. Thesis.

[66] SadAbadi H., Ghasemi M. (2007). Effects of some injection molding process parameters on fiber orientation tensor of short glass fiber polystyrene composites (SGF/PS). J. Reinf. Plast. Compos. Part A Appl. Sci. Manuf..

[67] Balla V.K., Kate K.H., Satyavolu J., Singh P., Tadimeti J.G.D. (2019). Additive manufacturing of natural fiber reinforced polymer composites: Processing and prospects. Compos. Part B Eng..

[68] Faudree M.C., Nishi Y., Gruskiewicz M. (2013). Characterization of Velocity Profile of Highly-Filled GFRP-BMC through Rectangular Duct-Shaped Specimen during Injection Molding from SEM Fiber Orientation Mapping. Mater. Trans..

[69] Liu X.C., Bathias C. (1993). Mechanical Properties of A1203/AI Composites. Metal Matrix Composites, Proceedings of the Ninth International Conference on Composite Materials (ICCM/9), Madrid, Spain, 12–16 July 1993.

[70] Pickering K.L., Efendy M.A., Le T.M. (2016). A review of recent developments in natural fibre composites and their mechanical performance. Compos. Part A Appl. Sci. Manuf..

[71] Li Y., Pickering K.L., Farrell R.L. (2009). Determination of interfacial shear strength of white rot fungi treated hemp fibre reinforced polypropylene. Compos. Sci. Technol..

[72] Vallejos M.E., Espinach F.X., Julian F., Torres L., Vilaseca F., Mutje P. (2012). Micromechanics of hemp strands in polypropylene composites. Compos. Sci. Technol..

